# Assessment of possible small fiber Neuropathy in early-stage vitamin B12 deficiency using electrophysiological methods

**DOI:** 10.1055/s-0045-1811722

**Published:** 2025-09-19

**Authors:** Elif Simin Issi

**Affiliations:** 1 Eskişehir City Hospital, Neurology Department, Eskişehir, Turkey.; 2 Afyonkarahisar Health Sciences University, Faculty of Medicine, Department of Neurology, Afyonkarahisar, Turkey.

**Keywords:** Vitamin B 12 Deficiency, Small Fiber Neuropathy, Galvanic Skin Response, Reflex, Abnormal, Electrodiagnosis

## Abstract

**Background:**

Small fiber neuropathy (SFN) affects thinly myelinated and unmyelinated fibers, often presenting with subtle clinical signs that are undetectable in routine nerve conduction studies. Vitamin B12 deficiency is a known risk factor for SFN, yet early-stage cases frequently remain undiagnosed. Sympathetic skin response (SSR) and cutaneous silent period (CSP) are noninvasive electrophysiological techniques used to assess autonomic and somatic small fiber function.

**Objective:**

The present study aimed to evaluate the diagnostic utility of SSR and CSP in detecting possible subclinical small-fiber neuropathy (pSFN) in individuals with early-stage vitamin B12 deficiency.

**Methods:**

The present observational study included 28 patients with vitamin B12 deficiency who had nonspecific complaints, Douleur Neuropathique en 4 Questions (DN4) scores < 4, and normal nerve conduction studies, along with 25 healthy controls. Electrophysiological testing involved SSR recordings from all extremities and CSP measurements from the right median and sural nerves.

**Results:**

In the patient group, Median Nerve Cutaneous Silent Period (MN-CSP) and Tibialis Anterior -Sural Cutaneous Silent Period (TA-sural CSP) durations were significantly shorter, while termination and onset latencies were prolonged compared with controls. MN-CSP and TA-sural CSP durations demonstrated high diagnostic accuracy. Sympathetic skin response latencies were significantly prolonged in both hands and feet, indicating autonomic dysfunction. No significant differences were observed in SSR amplitudes.

**Conclusion:**

Sympathetic skin response and CSP are valuable tools for detecting possible subclinical SFN in vitamin B12 deficiency. Sympathetic skin response effectively identified autonomic dysfunction, while CSP provided additional diagnostic value for somatic small fiber impairment. Combining SSR and CSP may enhance early detection of pSFN in vitamin B12 deficiency and allow timely intervention.

## INTRODUCTION

Vitamin B12 deficiency is a prevalent condition with significant neurological consequences worldwide. It can lead to various disorders affecting both the central and peripheral nervous systems. The most well-known neurological manifestations include sensory-motor axonal polyneuropathy and subacute combined degeneration, which may present symptoms such as muscle weakness, numbness, paresthesia, balance impairment, and gait disturbances. 1

Small fiber neuropathy (SFN), primarily affecting thinly myelinated A-delta and unmyelinated C fibers, can also result from vitamin B12 deficiency. Small fiber neuropathy is often characterized by sensory symptoms such as paresthesia, burning sensations, and pain. In addition to sensory dysfunction, SFN may involve autonomic nervous system impairment, leading to cardiovascular, gastrointestinal, and other systemic effects. Early diagnosis is crucial for effective management and to improve the quality of life of affected individuals. However, routine nerve conduction studies (NCS) may not detect early-stage SFN, requiring the use of more specialized diagnostic tools. 2

Noninvasive electrophysiological techniques play a critical role in the early diagnosis and monitoring of SFN. Günes et al. demonstrated through biopsy studies that both symptomatic and asymptomatic small fiber loss can occur in individuals with vitamin B12 deficiency. 2 This highlights the need for objective and reliable diagnostic methods for early detection.

The cutaneous silent period (CSP) is one such noninvasive electrophysiological test that assesses the function of thinly myelinated A-delta fibers. It is considered a sensitive tool for detecting SFN and monitoring disease progression. 3 4 5 The sympathetic skin response (SSR), another noninvasive method, is used to evaluate autonomic nervous system function. Compared to other autonomic electromyography (EMG) techniques, SSR requires minimal preparation, is quick to perform, and is a practical assessment tool. In conditions such as vitamin B12 deficiency, SSR serves as an auxiliary tool for evaluating autonomic nervous system functions and is useful in detecting autonomic dysfunction associated with small fiber impairment. 6

The present study aims to assess SFN using CSP and SSR measurements to facilitate the early diagnosis of neurological symptoms related to vitamin B12 deficiency. By evaluating these noninvasive electrophysiological techniques, we seek to demonstrate their effectiveness in detecting small fiber dysfunction before more severe symptoms develop.

## METHODS

The present observational, cross-sectional study was conducted in the Clinical Neurophysiology Department of Eskişehir City Hospital, Eskişehir, Turkey, between 2022 and 2024, with approval from the local ethics committee (ESH/BAEK 2024/20). Twenty-eight patients were enrolled after low serum vitamin B _12_ (< 190 pg/mL) was incidentally detected during routine laboratory testing and were subsequently referred for EMG with a preliminary diagnosis of polyneuropathy secondary to vitamin B _12_ deficiency. At presentation, each patient reported at least one mild, nonspecific complaint (e.g., headache, fatigue, subjective sensory discomfort). All had Douleur Neuropathique en 4 Questions (DN4) 7 scores < 4 and normal neurological examinations, indicating an absence of clinically overt neuropathic pain. The mean vitamin B _12_ level was 156.9 ± 20.4 pg/mL (range: 97–186). These inclusion criteria – DN4 < 4, normal large-fiber NCS, and planned SSR and CSP testing – were designed to capture early, clinically silent small-fiber involvement.

Exclusion criteria comprised diabetes mellitus, renal or hepatic failure, thyroid disorders, autoimmune disease, active infection, alcohol abuse, use of neurotoxic drugs such as chemotherapy agents, amiodarone, or statins, and any previously diagnosed central or peripheral nervous-system disorder. Alternative causes of small-fiber neuropathy were ruled out with complete blood count, HbA1c, renal, hepatic, and thyroid function tests, folate, vitamin D, lipid profile, erythrocyte sedimentation rate, and C-reactive protein. Methylmalonic acid and homocysteine measurements were unavailable at our center; this limitation is discussed later. Brain and spinal 3-Tesla magnetic resonance imaging (MRI) was performed in all patients, and no structural abnormalities were identified.

The control group consisted of 25 age- and sex-matched healthy individuals who attended the hospital for routine periodic health screening. All controls had serum vitamin B _12_ levels > 250 pg/mL and no systemic disease, neurotoxic medication use, or neurological symptoms.

Both groups underwent the following standardized assessment protocol: detailed neurological examination; motor nerve-conduction studies (median, ulnar, tibial, and peroneal nerves) and sensory studies (sural and superficial peroneal nerves); F-wave and tibial H-reflex analyses; sympathetic skin-response recordings from all four limbs; CSP measurements after median and sural stimulation; needle EMG when clinically indicated; and brain and spinal 3-Tesla MRI. In addition, a complete blood count—including mean corpuscular volume (MCV)—and a comprehensive biochemical panel (HbA1c, renal, hepatic and thyroid function tests, folate, vitamin D, lipid profile, erythrocyte sedimentation rate, and C-reactive protein) were obtained in every participant to exclude alternative causes of neuropathy. All electrophysiological, radiological, and laboratory findings lay within age-appropriate reference ranges in both patients and controls.

### Electrophysiological examinations

The present study was conducted in the Clinical Neurophysiology Unit of Eskişehir City Hospital, with all evaluations performed by the same specialist using a Natus Synergy on Nicolet EDX electromyography device (Natus). Recordings were taken between 10:00 AM and 12:00 PM in a quiet, well-lit, air-conditioned room maintained at a temperature of 24 ± 1°C. All participants were positioned comfortably in a supine posture, and their skin temperature was ensured to be > 32°C.

### Nerve conduction studies

Nerve Conduction Studies (NCS): Sensory NCS were done on right median, ulnar, radial, bilateral sural, and superficial peroneal nerves. Motor NCS were done on right median, ulnar and bilateral peroneal, and posterior tibial nerves.

### H-reflex and F-latency

Hoffmann reflex (H-reflex) and F-wave latency (F-latency) were assessed according to standard protocols. Right tibial nerve stimulation elicited the H-reflex in the soleus muscle and the F-latency response in the median and tibial nerves. The intensity for both measures was set to 1.5 times the sensory threshold, with a pulse width of 0.2 milliseconds (ms). Each pair of stimuli was delivered seven or eight times (preferably seven) for averaging. For soleus H-reflex recordings, a 10 ms train of stimuli was applied, incorporating three stimulation patterns with 1-second intervals between them. All final measurements represent the average of 10 trials. 8 9

### Cutaneous silent period measurements

Cutaneous silent period measurements were obtained by means of electrically stimulating the right index finger digital nerve (D2) and sural nerve, focusing on the abductor pollicis brevis (APB) and tibialis anterior (TA) muscles, respectively. Muscle activity was monitored while participants were comfortably positioned in a supine position, with visual and auditory feedback provided.

Stimulation was administered to the digital nerve of the right index finger for the abductor pollicis brevis muscle and to the sural nerve for the TA muscle. Electrodes were positioned in a belly-tendon arrangement. Electrical stimuli comprised rectangular pulses lasting 0.5 ms, administered at 8 to 10 times the sensory threshold, with a minimum interval of 10 seconds between stimuli.

Recordings were conducted with a low cutoff frequency of 30 Hz and a high cutoff frequency of 10,000 Hz. Each recording had a duration of 600 ms, comprising a 120-ms prestimulus (baseline) interval and a 480-ms poststimulus interval. The gain was established at 100–500 µV/division. 5

**Figure 1:**
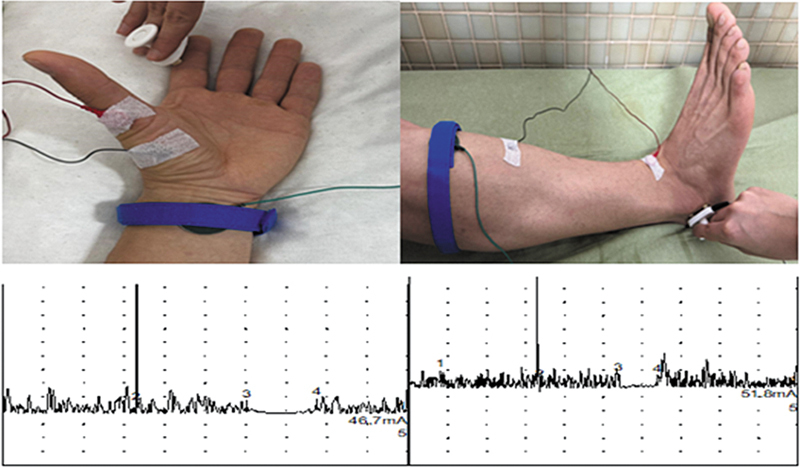
Cutaneous silent period recordings from the abductor pollicis brevis muscle (upper extremity) and tibialis anterior muscle (lower extremity). Abbreviation: CSP, cutaneous silent period.

Participants maintained ∼ 50% of their maximal isometric force during stimulation; recordings were paused if fatigue appeared. Silent-period onset latency and duration were measured in six trials and averaged for analysis ( Figure 1 ).

### Sympathetic skin responses

Recordings were obtained with a sweep speed of 10 seconds, sensitivity 500 µV, band-pass 0.2 to 100 Hz. Stainless steel disc electrodes with 6-mm diameter were used for recordings. The active electrodes were attached to the palmar and plantar surfaces of the hands and feet, and the reference electrodes were placed on the dorsal surfaces.

For SSR recordings at the palm level, electrical stimulation of the left median nerve at the wrist and for SSR recordings at the plantar level and electrical stimulation of the left posterior tibial nerve behind the medial malleolus were performed. Stimulation intensity was 25 mA, with a pulse duration of 0.2 ms; to minimize the risk of habituation, stimuli were applied with randomized intervals of 30 to 60 seconds.

Latency corresponded to the time interval between the stimulation artifact and the first moment of deflection from baseline. Amplitude was defined as the difference in distance from the onset of the negative deflection to the peak of the negative potential. 10

### Statistical analysis

Statistical analysis was performed with IBM SPSS Statistics for Windows (IBM Corp.) version 26.0. The Shapiro-Wilk test assessed normality of continuous variables. Normally distributed variables were compared with the independent samples t-test (Welch correction applied if the Levene's test indicated unequal variances) **;** non-normal variables would have been examined with the Mann-Whitney U-test, although this was not required for the key outcomes. Categorical variables such as sex were compared with the Pearson χ ^2^ test. Effect sizes were expressed as Cohen's d together with bias-corrected bootstrap 95% confidence intervals (CIs) (5,000 resamples). Linear associations between serum vitamin B _12_ levels and electrophysiological measures were assessed with two-tailed Pearson correlation; Spearman's ρ was used if either variable deviated from normality. A 2-sided *p* -value < 0.05 was considered statistically significant.

## RESULTS

When the demographic and anthropometric characteristics of the patient and control groups were compared, no significant differences emerged in gender, age, height, or body mass index (all *p* > 0.05). In contrast, serum vitamin B _12_ concentrations were markedly lower in the patient group (156.9 ± 20.4 pg/mL) than in controls (335.8 ± 103.8 pg/mL; *p* < 0.001; Cohen d = - 2.03; 95%CI: - 2.77–-1.25), while MCV was modestly but significantly higher (91.6 ± 8.6 fL versus 85.9 ± 5.5 fL; *p* < 0.0001; d = 0.78; 95%CI: 0.23–1.30). Macrocytosis (MCV > 96 fL) was present in 8/28 patients (2%) and in none of the controls ( Table 1 ). Routine nerve-conduction studies were within normal limits for all participants.

**Table 1:** Demographic and anthropometric characteristics of the study groups

Variable	Patients ( *n* = 28)	Controls ( *n* = 25)	*p-value* ^‡^
Age, years old	35.6 ± 13.4	35.4 ± 8.1	0.92
Male/Female, *n* (%)	9/19 (32/68)	10/15 (40/60)	0.76 ^†^
Height, cm	167.9 ± 9.8	169.5 ± 9.3	0.53
BMI, kg m ^−2^	25.6 ± 3.7	25.0 ± 5.1	0.68
Vitamin B _12_ , pg mL ^−1^	156.9 ± 20.4	335.8 ± 103.8	< 0.001
MCV, fL	91.6 ± 8.6	85.9 ± 5.5	< 0.0001
Macrocytosis (MCV > 96 fL), n (%)	8 (29)	0 (0)	0.001 ^†^

Abbreviations: BMI, body mass index; MCV, mean corpuscular volume.

Notes: Continuous variables are mean ± SD; categorical data are *n* (%). *P* -values from independent-samples t-test, except ^†^ χ ^2^ test for categorical comparisons; ^‡^ two-sided.

All 28 patients reported at least 1 mild, nonspecific symptom – headache (25%), fatigue (25%), forgetfulness (18%), musculoskeletal pain (14%), transient paresthesia/burning (11%), insomnia (4%), or tinnitus (4%) – yet none met neuropathic-pain criteria (DN4 < 4).

Electrophysiologically, median-nerve cutaneous silent-period (MN-CSP) duration was significantly shorter in patients than in controls (40.9 ± 11.7 ms versus 53.6 ± 13.5 ms; *p* = 0.001; Cohen's d = - 1.00; 95%CI: - 1.52–- 0.46). A comparable reduction was observed for tibialis-anterior/sural CSP (TA-sural CSP) duration (40.5 ± 10.4 ms versus 48.8 ± 10.5 ms; *p* = 0.004; d = - 0.79; 95%CI: - 1.29–- 0.27). In contrast, MN-CSP end latency and TA-sural CSP onset latency were longer in patients ( *p* = 0.010; d = 0.72; 95%CI: 0.15–1.26; *p* = 0.032; *d* = 0.56; 95%CI: 0.04–1.07, respectively) ( Table 2 ).

**Table 2:** Electrophysiological parameters and laboratory reference ranges

Parameter	Patients (mean ± SD)	Controls (mean ± SD)	*p-value*	Normal range	d (95% CI)*
Median CSP duration (ms)	40.9 ± 11.7	53.6 ± 13.5	0.001	27–81	- 1.00 (-1.52–-0.46)
Tibial CSP duration (ms)	40.5 ± 10.4	48.8 ± 10.5	0.004	28–70	- 0.79 (-1.29–-0.27)
Hand SSR latency (s)	1.40 ± 0.13	1.28 ± 0.12	0.002	≤ 1.52	0.99 (0.45–1.52)
Foot SSR latency (s)	2.03 ± 0.18	1.85 ± 0.16	< 0.0001	≤ 2.17	1.07 (0.51–1.60)
H-reflex latency (ms)	30.24 ± 2.19	28.99 ± 2.13	0.032	≤ 33	0.58 (0.05–1.09)

Abbreviations: CI, confidence interval; CSP, cutaneous silent period; ms, milliseconds; s, seconds; SSR, sympathetic skin response.

Notes: Values are mean ± SD; *p* from two-tailed *t* -test. Normal ranges derived from an independent cohort; within ± 2 SD of our lab mean and concordant with IFCN standards.

Sympathetic skin responses latencies were prolonged in patients for both the right foot (2.03 ± 0.18 s versus 1.85 ± 0.16 s; *p* < 0.001; d = 1.07; 95%CI: 0.51–1.60) and the right hand (1.40 ± 0.13 s versus 1.28 ± 0.12 s; *p* = 0.002; d = 0.99; 95%CI: 0.45–1.52), whereas amplitudes were comparable ( Table 2 ). H-reflex latency was modestly increased (30.24 ± 2.19 ms versus 28.99 ± 2.13 ms; *p* = 0.032; d = 0.58; 95%CI: 0.05–1.09).

All control subjects fell within our laboratory reference limits (MN-CSP 27–81 ms; TA-sural CSP 28–70 ms; hand-SSR latency ≤ 1.52 s; foot-SSR latency ≤ 2.17 s; H-reflex latency ≤ 33 ms). These cutoffs align with the recommendations of the International Federation of Clinical Neurophysiology (IFCN). Consequently, covert small-fiber neuropathy was effectively excluded in the control group. The consistently large Cohen's d values underline the clinical relevance of the somatic and autonomic small-fiber abnormalities observed in vitamin B _12_ -deficient patients.

## DISCUSSION

The present study demonstrates that SSR and CSP testing, when applied together, reliably detect subclinical SFN in early vitamin B _12_ deficiency. Prolonged SSR latencies signaled early autonomic C-fiber impairment, whereas markedly shortened CSP durations – accompanied by prolonged onset and end latencies – indicated somatic A-δ-fiber involvement. Large effect sizes – despite all individual values remaining within laboratory limits – underscore the clinical relevance of these group-level abnormalities.

Untreated cobalamin deficiency can progress to irreversible central and peripheral damage, most notably subacute combined degeneration of the spinal cord. Because serum B _12_ levels correlate poorly with clinical severity and ancillary biomarkers such as homocysteine or methylmalonic acid have limited accuracy, 11 objective functional tests are essential. Metabolic axonal injury first targets thinly myelinated A-δ and unmyelinated C fibers, producing neuropathic pain and autonomic symptoms before large-fiber involvement emerges. 12 13 14 15 By documenting shortened CSP and prolonged SSR latencies in otherwise asymptomatic individuals, our findings support incorporating these tests into the early diagnostic work-up of vitamin B _12_ deficiency.

Axonal or demyelinating neuropathy due to vitamin B _12_ deficiency is typically detected on nerve-conduction studies and EMG, which reveal prolonged distal latencies, low amplitudes, slowed velocities, and axonal loss. 11 12 Oral or intramuscular B _12_ replacement improves peripheral-neuropathy symptoms in 10 to 87% of patients and also lessens autonomic complaints and small-fiber dysfunction. 13 15

Numerous studies have demonstrated a clear association between vitamin B12 deficiency and autonomic dysfunction. Previous research has shown that reduced sympathetic and parasympathetic activity may result from vitamin B12 deficiency, and that these functions can be restored through replacement therapy. 16 17 18 Prolonged SSR latency and/or reduced amplitude may suggest demyelination or axonal damage in sympathetic pathways; however, SSR cannot localize the lesion precisely, and complementary tests (e.g., Quantitative Sudomotor Axon Reflex Test (QSART) or Heart Rate Variability (HRV) are required for definitive topography. Typically, demyelination is associated with delayed latency, whereas axonal damage leads to reduced amplitude. 5 10 19

Improvements in SSR latency and amplitude have been linked to the functional recovery of the sympathetic nervous system following B _12_ replacement therapy. 19 20 Consistent with previous work, our study showed significantly prolonged SSR latencies – most evident in the feet ( *p* < 0.001; Cohen's d = 1.07) – highlighting foot latency as a sensitive marker of early autonomic dysfunction even without predefined diagnostic cutoffs. Although these latencies remained within IFCN normal limits, their ≈ 1 SD right-shift relative to controls indicates subclinical slowing. Although SSR amplitudes were lower in the patient group than in controls, the difference was not statistically significant. This pattern suggests that latency changes may precede overt amplitude loss, indicating the earliest stage of demyelinating or axonal injury. Furthermore, vitamin B _12_ supplementation has been reported to improve autonomic parameters, including heart-rate variability, even in healthy individuals. 21 22 These observations support the use of SSR not only for early diagnosis but also for monitoring post-treatment recovery, while recognizing that SSR cannot localize the lesion precisely and should be interpreted alongside complementary tests. In this context, our findings confirm the potential of SSR to detect both pre- and postganglionic sympathetic dysfunction in vitamin B _12_ deficiency.

Cutaneous silent period, a marker of spinal inhibition, is especially sensitive to A-δ-fiber dysfunction. 3 5 In vitamin B _12_ deficiency, prolonged CSP latency suggests demyelination, whereas shortened duration indicates axonal loss from fiber dropout. 5 23 The pattern depends on disease length: acute deficiency chiefly causes axonal degeneration, while chronic deficiency leads to secondary demyelination, myelin loss, and some axonal regeneration. 23 24

In our study, both upper- and lower-extremity CSP measurements revealed significantly shortened MN-CSP and TA-sural CSP durations, demonstrating considerable diagnostic value for small-fiber neuropathy. Tibialis-anterior/sural CSP onset latency was also significantly prolonged in the patient group. Although these values remained within our laboratory reference ranges (MN-CSP: 27–81 ms; TA-sural CSP: 28–70 ms), the ≈ 1 SD right-shift relative to controls indicates subclinical involvement. The medium effect size for TA-sural latency (Cohen's d = 0.60; 95%CI: 0.04–1.07) further supports its practical value in early detection.

Similar to the prolonged SSR latencies observed, this finding may indicate an underlying demyelinating process. Conversely, the strong diagnostic performance of shortened CSP durations points toward axonal loss.

Post-treatment CSP gains show that B _12_ replacement benefits both demyelinating and axonal recovery. 5 25 Cutaneous silent period latency and duration track SFN and therapy response, while combining CSP with SSR can sharpen autonomic assessment.

Our results are in line with prior work showing the impact of vitamin B _12_ deficiency on SFN. 25 26 Histopathological studies have demonstrated reduced intraepidermal nerve-fiber density even in asymptomatic B _12_ -deficient individuals, 2 indicating that nerve damage may begin before overt symptoms appear.

In our study, patients presenting with nonspecific complaints and DN4 scores < 4 exhibited early signs of SFN. Sympathetic skin responses and CSP were found to be effective in detecting early small fiber dysfunction. Foot-recorded SSR latencies separated patients from controls with a large effect size (Cohen's *d* ∼ 1.1), underscoring their value as early markers of autonomic impairment.

Prolonged tibial H-reflex latencies provide additional evidence of subclinical involvement of large myelinated fibers; when F-wave latency is normal, this delay is attributed chiefly to conduction slowing along the most distal segment of the Ia-afferent/α-motor loop, a recognized electrophysiological marker of length-dependent (distally predominant) neuropathy. 9 27

Cutaneous silent period measurements also revealed significant findings. Both MN-CSP and TA-sural CSP durations exhibited large effect sizes (Cohen's *d* ≥ 0.8), underscoring their high clinical utility. A shortening of CSP duration typically reflects axonal loss, whereas a prolonged latency is more consistent with demyelination. 5 25

The combined use of SSR and CSP enables a comprehensive evaluation of both autonomic and somatic small-fiber involvement, highlighting their complementary clinical roles. 19 25

The present study shows that SSR and CSP can reveal probable sub-clinical small-fiber dysfunction in early-stage vitamin B _12_ deficiency. Its main limitations are:

- The modest sample size;
- Case definition based solely on serum B _12_ concentration, because cost- and logistics-related constraints prevented measurement of functional biomarkers such as methylmalonic acid or homocysteine;
- Absence of gold-standard confirmation of small-fiber loss (skin biopsy or quantitative sensory testing);
- Lack of post-treatment follow-up;
- The inherent susceptibility of SSR to habituation and intersession variability despite stimulus randomization.

Addressing these issues in future, larger cohorts—ideally with histological or Quantitative Sensory Testing [QST] validation and longitudinal reassessment after B _12_ replacement—will clarify the full clinical utility of SSR and CSP.

In conclusion, the present study underscores the utility of SSR and CSP as complementary, noninvasive tools for evaluating autonomic and somatic small-fiber dysfunction in early-stage vitamin B _12_ deficiency. Sympathetic skin response capture dysfunction of sympathetic unmyelinated C fibers but, by itself, cannot precisely localize the lesion within the autonomic pathway, while CSP reflects A-delta fiber impairment, enabling the identification of small-fiber pathology even in patients with nonspecific or subclinical symptoms. Incorporating these techniques into routine clinical practice may facilitate earlier diagnosis and allow objective monitoring of treatment response. Future studies with larger cohorts, posttreatment assessments, and the addition of functional biomarkers such as Methylmalonic Acid (MMA) and homocysteine are warranted to enhance diagnostic accuracy and clinical applicability. Establishing normative values across different age groups and populations will further support personalized management strategies in B _12_ -related neuropathies.
